# Occurrence and environmental data for aquatic plants of Minnesota from 1999–2018

**DOI:** 10.1038/s41597-026-07027-3

**Published:** 2026-03-11

**Authors:** Michael R. Verhoeven, William L. Bartodziej, Matthew S. Berg, Simba Blood, Rachael Crabb, Eric Fieldseth, James A. Johnson, Jimmy Marty, Steve McComas, Raymond M. Newman, Meg Rattei, Jill B. Sweet, Justin Townsend, Brian Vlach, Justin Valenty, Jerry P. Spetzman, Susanna W. Witkowski, Andrea Prichard, Wesley J. Glisson, Daniel J. Larkin

**Affiliations:** 1 Department of Fisheries, Wildlife, and Conservation Biology & Minnesota Aquatic Invasive Species Research Center, University of Minnesota – Twin Cities, Saint Paul, USA; 2 Ramsey-Washington Metro Watershed District, Canada, USA; 3 Endangered Resource Services, LLC, Greensboro, USA; 4 Minneapolis Park & Recreation Board, Minneapolis, USA; 5 AIS Consulting Services, London, UK; 6 Freshwater Scientific Services, Osseo, USA; 7 Emmons & Olivier Resources, Inc, St Paul, USA; 8 Blue Water Science, Cincinnati, USA; 9 Barr Engineering, Minneapolis, USA; 10 Minnehaha Creek Watershed District, Minnetonka, 55345 USA; 11 Ramsey County Parks and Recreation Soil and Water Conservation Division, Roseville, USA; 12 Three Rivers Park District, Plymouth, USA; 13 Chisago County Environmental Services, Center City, USA; 14 Present Address: Washington State Department of Ecology, Lacey, USA

**Keywords:** Freshwater ecology, Invasive species, Biodiversity, Conservation biology, Biodiversity

## Abstract

The aquatic flora of Minnesota’s freshwater lakes have been extensively surveyed for purposes of resource assessment, research, and ecosystem management. Despite widespread use of a common method for vegetation sampling (“point-intercept surveys”), these records have existed to-date in disparate locations without unification. Here we present a first-of-its-kind dataset of point-level occurrences, relative abundances, and associated environmental data for macrophytes (freshwater plants) across Minnesota. The data encompass 3,194 surveys of 1,520 lakes and ponds performed over a 19-year timespan. A total of 367,382 points were sampled, across which 231 taxa were recorded. Macrophyte occurrence data and depth, as well as point-level relative-plant-abundance measures for a subset of surveys, were collated, cleaned, and joined to geospatial data and Secchi-depth measurements of water clarity, enabling light availability, a primary control on aquatic plant growth, to be estimated. The data are well-suited for ecological analyses across multiple spatial scales and can be used to address both fundamental and applied ecological questions.

## Background & Summary

Monitoring aquatic plant communities is a primary means of understanding ecosystem condition and change in the world’s freshwaters^1–4^. At local scales, monitoring is motivated by efforts to assess, manage, and restore waterbodies^5,6^. At broad scales, occurrence data have been used to understand macroecological relationships and fundamental ecology^7,8^. When aquatic plant surveys are collected for each of these purposes, they are often equally useful for other research or management needs: One survey can serve multiple purposes, ranging from guiding adaptive management, to assessing status and trends of threatened systems, to elucidating the evolutionary and ecological underpinnings of species distribution and function. However, if these data are not collated and made accessible to others, they only serve the narrower purposes and stakeholders for which they were originally collected^9^. This is an unfortunate gap given that many questions remain to be answered about macrophyte ecology and management. For example, some questions about management effects might be readily addressed by increased availability of data that could serve as unmanaged reference systems^10^, and other macrophyte biogeography questions could be answered by increased availability and interoperability of local sampling. Questions remain about the macroecological patterns of aquatic plants, and these data are especially well suited to assessing pattern changes across scales, incorporating explicit abundance estimation, and evaluating temporal changes^7,11–13^.

Early studies on aquatic plants largely consisted of observational studies with inconsistently applied or biased methods and could not be used for reliable abundance estimation^1,14^. However, in the U.S. over the last two decades, a standardized aquatic plant survey methodology has been widely adopted—the rake-based point-intercept survey^15,16^. This has resulted in consistently collected, spatially explicit data collected by many surveyors over large geographic areas. The point-intercept survey framework, as applied in freshwater plant systems, enables statistical estimation of uncertainty and accounting for key sources of bias^10,15–17^.

The utility of these plant community data for answering questions beyond the scale of a single project or waterbody are typically limited by the data being collected, analyzed, and stored by small groups of end users^18,19^. When efforts have been made to collate such data, the resulting syntheses have proven valuable for advancing both theoretical and applied questions in plant ecology^1,10,12,13,20^. Although directly comparable data now exist (as rake-based point-intercept surveys) for aquatic plant communities across broad regions, barriers including disparate storage locations, curation, and lack of data standardization have meant that researchers lack ready access to these data—or even knowledge of their existence. The centralized, open-access dataset presented here is intended to support foundational studies of aquatic plant ecology and help guide adaptive management by collating and thoroughly describing these data. Indeed, these data have already seen use in evaluations of macrophyte ecology in Minnesota^10,21–24^. Subsequent work should continue to develop a means by which this database can be readily updated with additional data collected and stored by the wide array of surveyors conducting work in Minnesota and beyond.

**Fig. 1:**
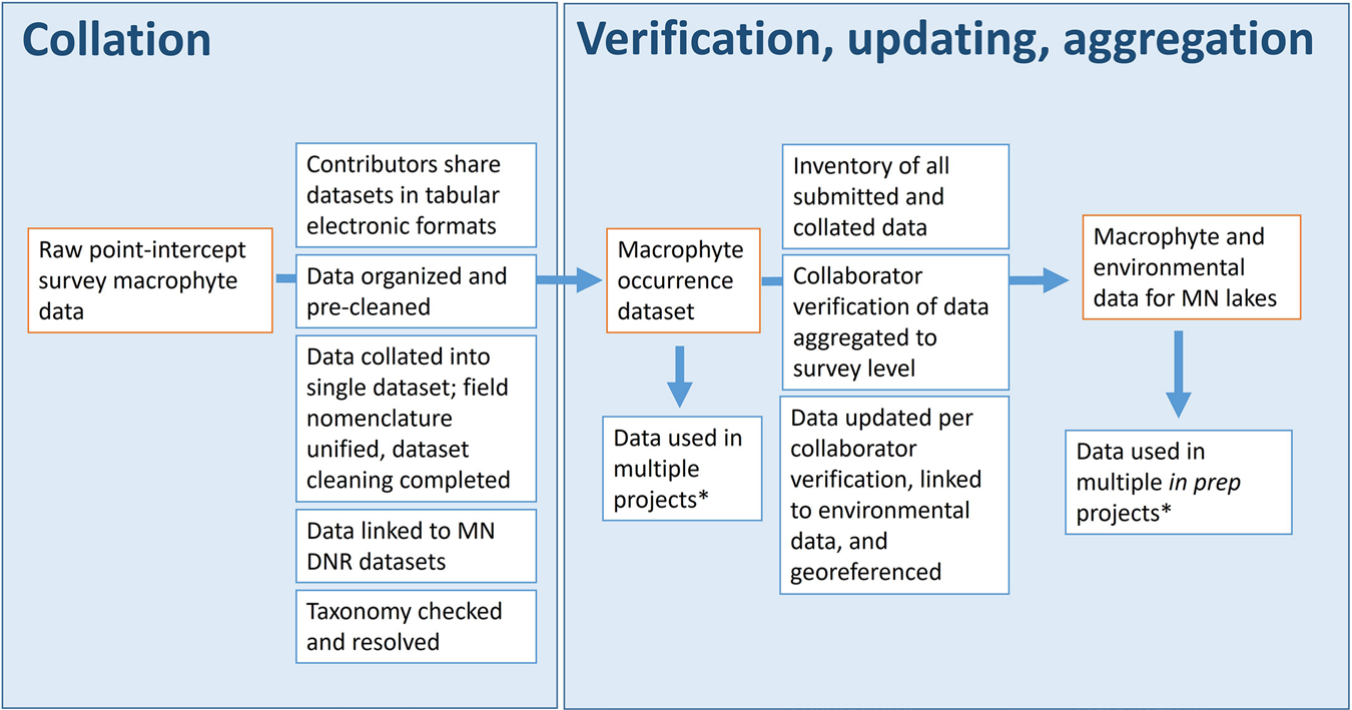
Macrophyte survey data collation, verification, and aggregation workflow. Orange boxes indicate data products, blue boxes indicate processes applied to those products. In collation phase, MN DNR datasets refers to incorporation of Minnesota Department of Natural Resources point-intercept data into the database. *Projects are included in references section^10,21–24^.

**Fig. 2:**
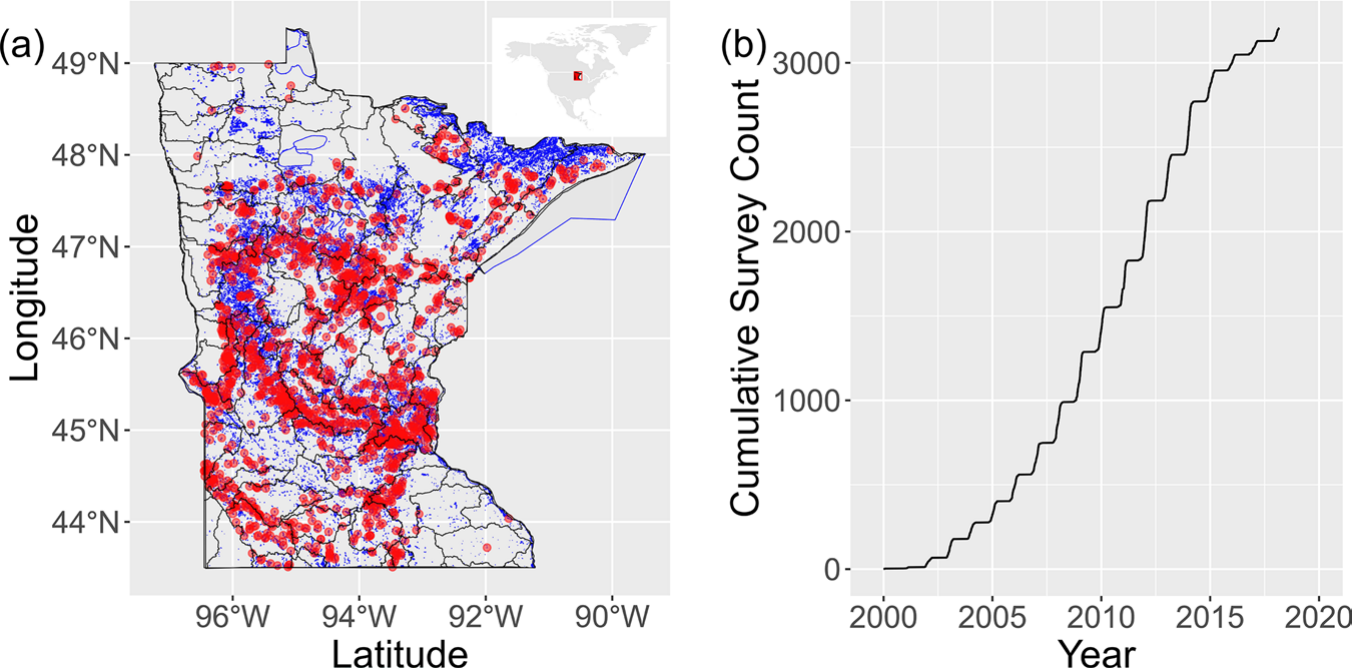
Spatial distribution (**a**) and temporal accumulation from 2000–2018 (**b**) of surveys in the dataset of aquatic plants across Minnesota, USA. For (**a**), lakes in MN are shown in blue, major watersheds (8 digit Hydrologic Unit Code) outlined in black, and red dots indicate lakes with one or more aquatic plant survey in the dataset.

We identified, acquired, collated, and aggregated to summarize (Fig. 1) aquatic plant monitoring data that were collected in the state of Minnesota (USA) over a 19-year period (2000–2018; Fig. 2). Data were compiled into a single dataset at the observation level (individual survey point) then cleaned and verified by contributors for quality, completeness, and taxonomic consistency. The datasets produced and associated scripts and metadata can be downloaded freely from the University of Minnesota Data Repository^25^.

## Methods

The datasets presented here were generated by in two phases. We began by procuring data in raw formats from collaborators and stakeholders, then collating and cleaning data to create a unified occurrence dataset. Next we verified collation and cleaning through inventory and evaluation of an intermediate data product, and joined records to existing environmental and spatial data sets. The final data are also aggregated at the point-, survey-, and watershed-level to produce multi-scale summary statistics and data products (Fig. 1).

### Plant observations

The observations level dataset comprises observations of aquatic plants by surveyors trained in macrophyte identification implementing rake based point-intercept surveys^16^. This included both previously published datasets^13,26^ and many surveys not previously published elsewhere.A primary advantage of the point intercept sampling scheme and its derivations is that the pre-fieldwork survey design yields samples that are spatially unbiased and treatment of these samples as proportional occupancy allows for direct comparison across surveys of differing effort. *A priori* determination of points to be sampled; both blindly systematic and spatially random point placement were used in the surveys in the data, and as a result, point-intercept survey data meet assumptions of unbiased sampling for statistical modeling. In addition, sampling is conducted such that the effort at each point is consistent: a surveyor uses a 0.33-m wide doubleheaded metal rake or a grappling hook style rake to drag approximately 3 m along the benthos^15–17,27,28^. The vegetation that becomes entangled in the rake is then identified to the lowest taxonomic level possible. Although this method does over- and under-sample certain species (e.g., dragged rakes over-sample *Hydrilla verticillata* biomass, under-sample *Vallisneria americana* presence), these biases are inherent to the method and generally apply uniformly across all samples (e.g., among surveyors, seasonal timing, etc.)^16,29–33^. To constrain the data to directly comparable records, we included only surveys conducted via a point-intercept design with sampling via a dragged-rake methodology (vs. a “spun-rake” method, hydroacoustics, SCUBA quadrat sampling, etc.).

We requested and collected any such point intercept macrophyte datasets from lake management entities across Minnesota that were available in a digital, tabular format (e.g., ArcGIS .dbf, Microsoft Excel, .csv, etc.). In total, 18 entities contributed data, and the contributing entity corresponding to data in the presented files can be found under the field “SURVEY_DATASOURCE” of the data products presented here. Data collected via congruent methods but submitted in unusable formats (e.g., hard copy, without point-level data) or that failed to integrate via our semi-automated processing were documented in the dataset, but their observation-level data were not included (257 surveys).

Data were collated through a partially automated process. All surveys from an individual data contributor were first compiled into a single dataset with a row for each point. Each contributor’s dataset was then merged into the primary dataset. At each of these steps, we accumulated species observations recorded as common names or surveyor-specific codes. We translated these to scientific names using keys provided by the data contributors or referencing common names in regional identification guides^34–36^.

For a subset of surveys, surveyors also assigned to each species at each sampling point a visual rake fullness score as an indicator of relative abundance^27,28,33^. These ordinal scales vary among surveyors as ranges from 1 (small amounts of a given species) to 3, 4, or 5 (rake fully covered by species). We normalized all scales to a common 1–3 standard; this was done by setting the endpoints of all scales to 1 and 3, then lumping all mid-scale values to values of 2. As a result, the endpoints of these abundance data (1 and 3) describe relatively specific conditions of rake coverage, while the middle value (2) encompasses a broad range of intermediate values. In addition, the absence of a species is often recorded as blank, NA, or zero—we converted all absences to zeros in the data which assumes that species not noted were not present in a sample.

### Depth

We only included observations in our dataset for which surveyors recorded water depths (via hydroacoustics, measuring poles, or sounding lines). We omitted sample points with a depth of 0 or NA to exclude shoreline/terrestrial observations and unsampled locations. Submitted depth data were in meters or feet but sometimes omitted the units used. Where units were not specified, we plotted depth × vegetation-distribution curves to determine the units based on typical colonization depths for plants. Where this approach was inconclusive, we compared maximum observed depth to lake bathymetry data to determine units (e.g., if maximum survey depth was 15, and the maximum depth of the waterbody was 5 m (16.4 feet) the measurement units were determined to be feet). Finally, if neither of these methods resolved ambiguity, other surveys conducted by the same surveyors/entities in the same year (where applicable) were used to determine depth units or the records were discarded.

### Water clarity

Water clarity data were added to the observations dataset as Secchi-depth observations at the lake or waterbody level. Secchi depth observations were acquired directly from the authors of an independent dataset compiled for research on lake clarity^37^. Because Secchi observations were not collected simultaneously with plant and depth data, we joined each observation record to the temporally closest Secchi observations within +/−1 year of the plant observation from the waterbody. The Secchi measurement and its date are included in the dataset and parameters for the join can be adjusted in the script available in the data repository. For each observation where Secchi data were available, we used water and Secchi depths to calculate light availability at the substrate^21^.

### Spatial locations

Lake centroid coordinates (latitude and longitude) are included for all data submitted with waterbody identifiers using Minnesota Division of Waters codes (unique codes assigned to public waterbodies of Minnesota). We additionally incorporated point-level location data when they were included with the submission, retaining information on coordinate systems or projections used and finally converted all spatial data to units of latitude and longitude. In summary, where sample point coordinates were submitted to us, they are available in the dataset; where location of a survey was described at the waterbody level we have included the centroid of that waterbody in the dataset; where we were unable to identify the waterbody from which survey data were collected the survey’s data remain in the dataset without any spatial information (18 surveys). After georeferencing data to Minnesota Department of Natural Resources waterbody geodata^38^, we connected plant observations to the major watershed in which their lake centroid falls (8 digit hydrologic unit code level, hereafter HUC-8; similar methods could be used to summarize at any spatial scale of interest) using Minnesota Department of Natural Resources watershed geodata^39^. For each watershed, we then summarized the plant occurrence data as outlined below.

### Taxonomy

**Fig. 3:**
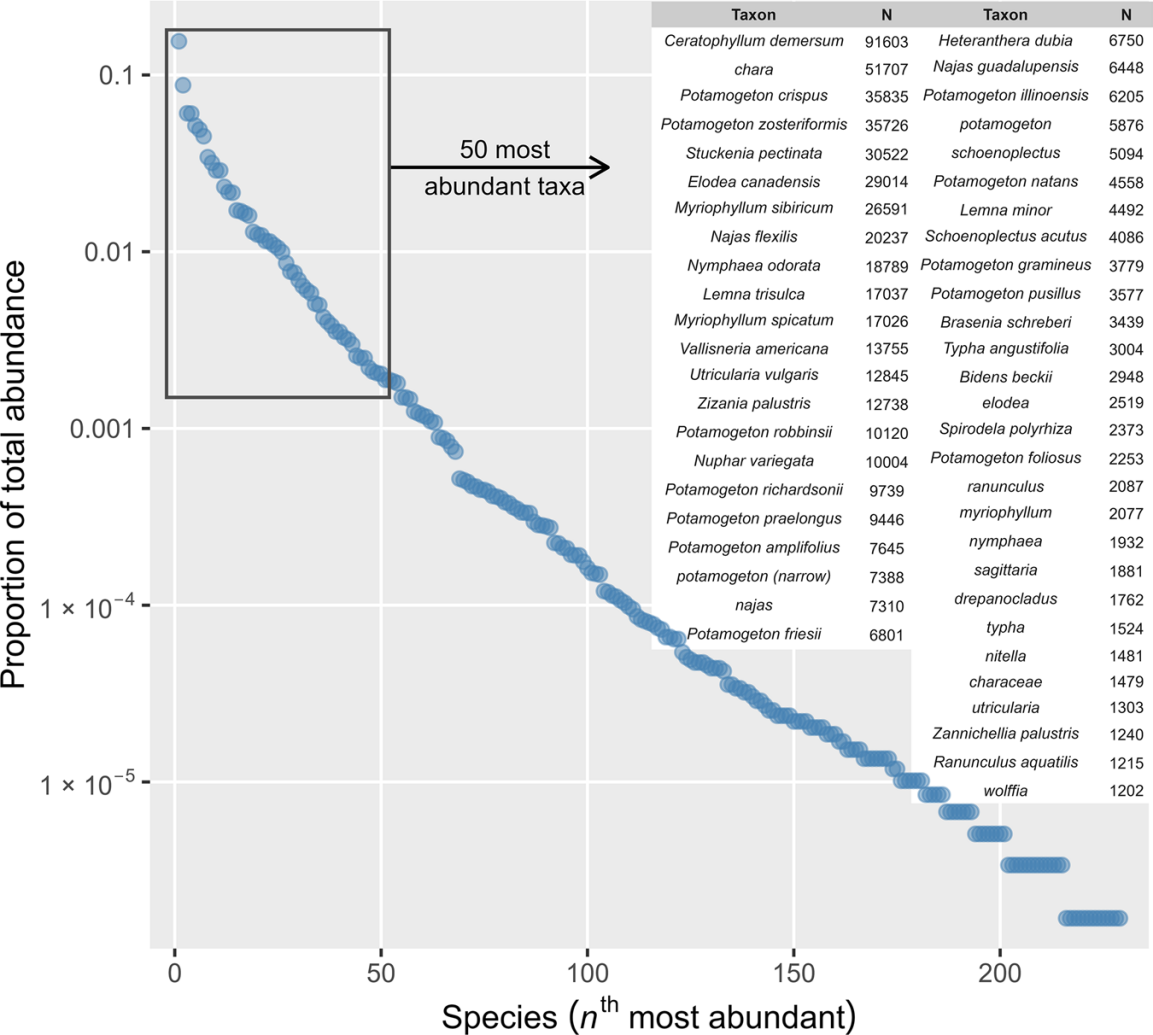
Taxon rank abundance distribution and counts of observations for the 50 most commonly observed taxa. Here, rank abundance and counts are calculated from point observations, but the dataset also allows for similar calculations to be made at lake and watershed scales.

As surveys were collated, we accumulated many observations of taxa that were recorded as common names or surveyor-specific codes. These were translated to scientific names using keys provided by the data contributors or referencing common names in regional identification guides^34–36^. Nomenclature was finalized and synonymy resolved based on the Taxonomic Name Resolution Service accessed via the *taxize* package in R^40,41^, followed by manual review applying regional knowledge of aquatic flora. In total, 231 taxa were retained in the dataset, including species, genus, and family level observations (Fig. 3). In compliance with state regulations aimed at conserving protected species in Minnesota, we have removed the identities of protected species. Using the Minnesota Department of Natural Resources’ “plant checklist”^42^ we distinguished native taxa from those with non-native status in Minnesota and complete data summarizations steps for all taxa and for native taxa only (e.g., calculated both total taxa richness, and native taxa richness). If a species’ status changes in the future, the code can be edited to reflect these changes.

### Data summarization

To complement our observation-level data, code in the repository calculates survey-level (waterbody × sampling instance) summary statistics commonly used to assess aquatic plant communities. These include various metrics describing depths sampled and depths with vegetation present, as well as plant community diversity and richness. For each survey, we include number of observations per taxon, which can be used to generate multiple variants of *frequency of occurrence*, for example by dividing by the total number of points sampled, the number of vegetated points, or the total number of points within the maximum depth ever observed with vegetation for that waterbody. Each has advantages, and should be chosen depending on the needs of the user (e.g, unbiased estimates across surveys with heterogeneous sampling depths can be attained by consistent handling of this divisor through time^10^).

At point, survey, and watershed scales, we calculated diversity as the Effective Number of Species based on Probability of Interspecific Encounter(${{ENS}}_{{PIE}}$) (Table 1, Fig. 3). This metric is robust to scale-dependent anomalies of diversity data^43^. ${{ENS}}_{{PIE}}$ is equivalent to inverse Simpson’s diversity, or:

$${{ENS}}_{{PIE}}=\frac{1}{\mathop{\sum }\limits_{i=1}^{S}\,{{p}_{i}}^{2}}$$

**Fig. 4:**
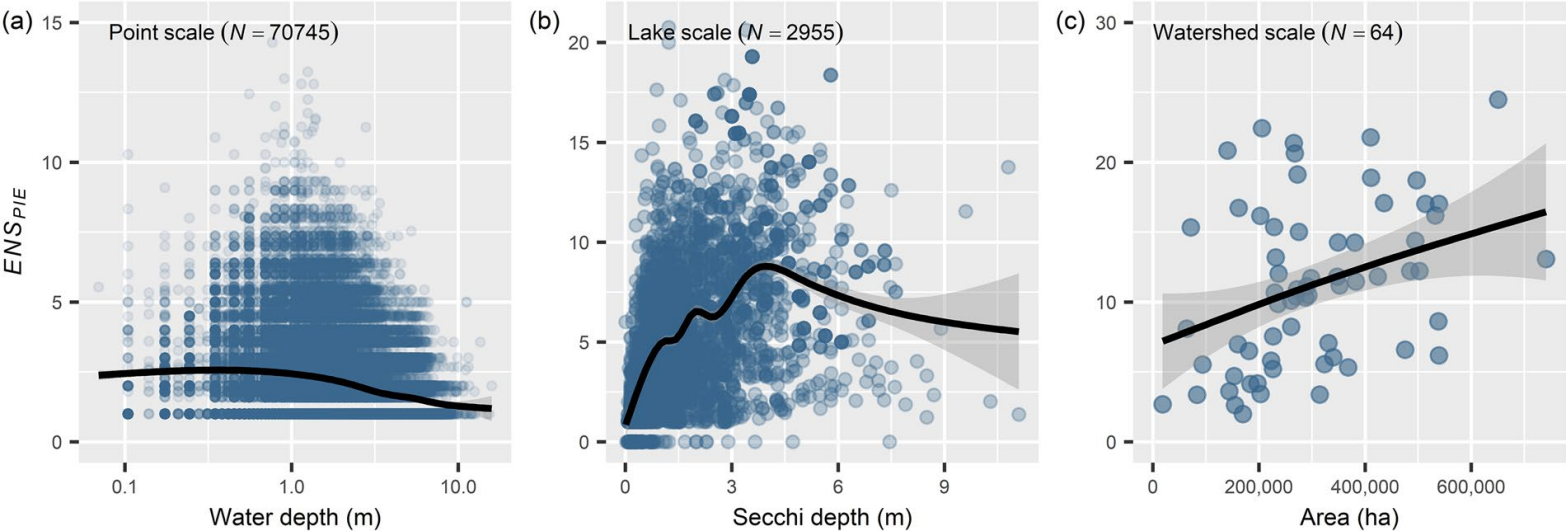
Environmental covariates and diversity metrics are available across all scales in the dataset. Here ENSpie (Inverse Simpson’s Diversity) calculated at three scales is plotted against three unique environmental variables. Black lines represent an automatic smoothed generalized additive model fit by *mcgv*::gam()^44^.

Where, $S$ is the total number of species and ${p}_{i}$ is the proportional abundance of species $i$. We also calculated species richness and Shannon’s diversity index, at multiple scales. These calculations are thus available for multiple scales of the data and can be readily retrieved from the data products (Fig. 4).

**Table 1:** Correlations (Pearson’s *r*) between native plant community response metrics for each scale analyzed.

Median Unit Area (m^2^)	Spatial Study Unit	n	r: ENS_PIE_-Richness	r: ENS_PIE_-Evenness	r: Richness - Evenness
2.72 × 109	Watershed	64	0.779	0.222	−0.264
6.40 × 105	Lake	2955	0.855	−0.201	−0.548
1	Point	70745	0.992	−0.388	−0.489

## Data Records

Static versions of the dataset at the observation level (plants_env_data.csv), point level (plants_env_data_wide.csv), the subset of point level with abundance data (plants_abund_env_data_wide.csv) and aggregated to the survey level (surveys_aqplants.csv; missing_data_surveys.csv) and watershed level (watershed_occurrence_wide.csv) are available from the Data Repository for University of Minnesota^25^ (Table 2). A complete metadata record is included and describes the variables in the data products, and the processing script. Code to complete data verification, updating and aggregation is also included in the repository as both an R script and an .html report version of that script.

**Table 2:** Data products associated with this data release.

Data Type	Data unit (row)	Dimensions (columns × rows)	filename
Plant observations	Taxa observation	30 × 726,923	plants_env_data.csv
Point presence/absence	Community presence/ absence at a point	251 × 367,382	plants_env_data_wide.csv
Point abundances	Community rake abundances at a point	165 × 118,868	plants_abund_env_data_wide.csv
Survey abundances	Survey of plant community summary	321 × 3,614	surveys_aqplants.csv
Surveys missing plant data	Survey of a plant community	23 × 257	missing_data_surveys.csv
Watershed plant observations	Watershed plant community summary	236 × 68	watershed_occurrence_wide.csv

## Technical Validation

The data were collated from many sources, including some previously published studies. All data are provided with reference to their source, including citations when applicable. In addition to the steps described above, all data contributors reviewed survey level summaries of the data they contributed to the project to check for errors in data entry or aggregation processes. The feedback provided by this verification process was then used to update the dataset (Fig. 1). The corrections can be viewed in the processing script available in the data repository.

## Usage Notes

Where surveys were identified, but no observation-level data have been submitted to this project, the survey date and location are included (missing_data_surveys.csv) for the benefit of others who may wish to seek out these records (e.g., stakeholders involved in management of a given lake).

Some surveys in the dataset were of small extents, encompassing only certain bays or subunits of a lake (common in large lakes with complex shorelines) or only certain depth strata (common in lakes with large extents that are too deep to support rooted vegetation). The former consideration has been handled in the data by identifying these surveys using a “subbasin” field. Care should be taken to ensure that a sub-lake survey is not taken as representative of a whole-lake’s plant community. Next, sampling extents limited by depth strata are not explicitly treated in the datasets. Instead, when the data are used, this limitation must be considered and accounted for if it will have an effect on the outcome of the analysis or use of the data^10^. Based on the application of the data, further cleaning of plant occurrences based on depths is recommended. Because multiple sample points are located within each depth stratum, confidence in survey findings can be estimated for each depth stratum using the number of sampled locations as “attempts” and occurrences of a taxon of interest as “successes” in a binomial error distribution^10^.

## Data Availability

All data from the verification, updating and aggregation phase (Table 1) of this work are available from the Data Repository for University of Minnesota^25^. Raw data contributed by individual contributing organizations are available on request from the authors.

## Code availability

Data aggregation, processing, and analyses to develop the datasets were all conducted in R version 4.2.0 (R Core Team, 2022). A processing script (a_Mnsynthesis_dataprep.R) and an html report generated from that script (a_Mnsynthesis_dataprep.html) can be used to reproduce the preparation and summarization of the data for this publication (Fig. 1-Verification, updating, aggregation). Both are available from the Data Repository for University of Minnesota^25^.
